# Histochrome Attenuates Myocardial Ischemia-Reperfusion Injury by Inhibiting Ferroptosis-Induced Cardiomyocyte Death

**DOI:** 10.3390/antiox10101624

**Published:** 2021-10-15

**Authors:** Ji-Won Hwang, Jae-Hyun Park, Bong-Woo Park, Hyeok Kim, Jin-Ju Kim, Woo-Sup Sim, Natalia P. Mishchenko, Sergey A. Fedoreyev, Elena A. Vasileva, Kiwon Ban, Hun-Jun Park, Sang-Hong Baek

**Affiliations:** 1 Department of Biomedicine & Health Sciences, The Catholic University of Korea, Seoul 06591, Korea; jidnjs2@catholic.ac.kr (J.-W.H.); hbreaks808@catholic.ac.kr (J.-H.P.); bwpark@catholic.ac.kr (B.-W.P.); hyeokkim@catholic.ac.kr (H.K.); wlswn0338@catholic.ac.kr (J.-J.K.); woosup269@catholic.ac.kr (W.-S.S.); 2 G.B. Elyakov Pacific Institute of Bioorganic Chemistry, Far-Eastern Branch of the Russian Academy of Science, 690022 Vladivostok, Russia; mischenkonp@mail.ru (N.P.M.); fedoreev-s@mail.ru (S.A.F.); vasilieva_el_an@mail.ru (E.A.V.); 3 Department of Biomedical Sciences, City University of Hong Kong, Kowloon Tong 999077, Hong Kong; 4 Department of Medical Life Science, College of Medicine, The Catholic University of Korea, Seoul 06591, Korea; 5 Division of Cardiology, Department of Internal Medicine, Seoul St. Mary’s Hospital, The Catholic University of Korea, Seoul 06591, Korea

**Keywords:** histochrome, iron-chelating, ischemia-reperfusion injury, anti-ferroptosis, cardioprotection

## Abstract

Reactive oxygen species (ROS) and intracellular iron levels are critical modulators of lipid peroxidation that trigger iron-dependent non-apoptotic ferroptosis in myocardial ischemia-reperfusion (I/R) injury. Histochrome (HC), with a potent antioxidant moiety and iron-chelating capacity, is now available in clinical practice. However, limited data are available about the protective effects of HC on ferroptotic cell death in myocardial I/R injury. In this study, we investigated whether the intravenous administration of HC (1 mg/kg) prior to reperfusion could decrease myocardial damage by reducing ferroptosis. Rats undergoing 60 min of ischemia and reperfusion were randomly divided into three groups as follows: (1) Sham, (2) I/R control, and (3) I/R + HC. Serial echocardiography up to four weeks after I/R injury showed that intravenous injection of HC significantly improved cardiac function compared to the I/R controls. In addition, the hearts of rats who received intravenous injection of HC exhibited significantly lower cardiac fibrosis and higher capillary density. HC treatment decreased intracellular and mitochondrial ROS levels by upregulating the expression of nuclear factor erythroid 2-related factor (*Nrf2*) and its downstream genes. HC also inhibited erastin- and RSL3-induced ferroptosis in rat neonatal cardiomyocytes by maintaining the intracellular glutathione level and through upregulated activity of glutathione peroxidase 4. These findings suggest that early intervention with HC before reperfusion rescued myocardium from I/R injury by preventing ferroptotic cell death. Therefore, HC is a promising therapeutic option to provide secondary cardioprotection in patients who undergo coronary reperfusion therapy.

## 1. Introduction

Acute myocardial infarction (AMI) is the leading cause of death and critical disability worldwide. The detrimental effects of AMI are primarily associated with the extent of cell dysfunction, injury, and/or death, which are substantially affected by both the magnitude and the duration of ischemia [1,2,3,4]. Currently, the most effective therapeutic strategy to diminish ischemic injury and infarction size is timely myocardial reperfusion using either anti-thrombosis therapy or primary percutaneous coronary intervention (PCI). However, although the occluded artery is fully opened with primary PCI, the process of myocardial reperfusion itself can induce further cardiomyocyte (CM) death by ischemia-reperfusion (I/R) injury [5,6]. Therefore, the strategy to reduce the extent of I/R injury is a promising therapeutic option to prevent adverse remodeling and worse clinical outcomes post-MI (Myocardial infarction) [7].

Reactive oxygen species (ROS), such as hydroxyl radicals (OH^•−^) and superoxide anions (O_2_^•−^), are the major mediators of myocardial I/R injury. While ROS remain at optimal concentrations under homeostatic balance, they can play a role as second messengers to control several cellular functions, such as cell survival, growth, and differentiation [8]. However, when adequate redox homeostasis is compromised due to excessive ROS levels and decreased antioxidant activity, it can cause DNA damage, protein denaturation, and lipid peroxidation [9], which might lead to various programmed cell death processes [10,11]. Although many cardioprotective strategies against I/R injury have been proposed according to their cellular and intracellular targets [12,13,14], their translations into the clinical setting have been disappointing [15], without patient benefit, partly because these strategies focus on caspase-dependent apoptosis as a drug mechanism and target [16].

Ferroptosis is non-apoptotic programmed cell death that is characterized by iron-dependent lipid peroxidation [17]. The morphological features of ferroptotic cell death appear as shrunken mitochondria with increased membrane density or outer membrane rupture. This distinctive modality of cell death has been appreciated in many pathological processes such as cancer, Parkinson’s disease, and stroke [18,19,20]. Myocardial I/R injury is also characterized by iron deposition as well as excessive ROS production, which might favor the induction of ferroptosis. Emerging evidence has suggested that ferroptosis occurred in transplanted I/R hearts, and the inhibition of ferroptosis resulted in reduced infarct size, improved left ventricular (LV) systolic function, and reduced LV remodeling [21,22]. Therefore, ferroptotic cell death [11] is an attractive therapeutic target against myocardial I/R injury, and identifying potential therapies is critical to preventing secondary myocardial damage after coronary reperfusion [21,23,24].

Echinochrome A (Ech A) is a lipophilic and membrane-permeable dark-red pigment in sea urchin shells and spines [25]. Histochrome (HC) is a water-soluble form of Ech A [26] and has strong antioxidant [27,28] and iron-chelating effects [29,30,31]. This drug is already used in Russia commercially as a 1% HC solution given via boluses or droppers and showed significant cardioprotective effect against I/R injury by reducing infarct size and arrhythmia potential [32,33,34]. However, limited data are available about the mechanism of HC focused on the ferroptotic cell death in myocardial I/R injury. In this study, we hypothesized that the intravenous administration of HC (1 mg/kg) [26,32] immediately before reperfusion could decrease myocardial cell death by reducing ferroptosis, which might improve cardiac function and remodeling in I/R-injured hearts.

## 2. Materials and Methods

### 2.1. Histochrome

Standardized echinochrome A (registration number in the Russian Federation is P N002362/01) was isolated from sea urchin Scaphechinus as described [35], and the purity of echinochrome A (99.0%) was confirmed by liquid chromatography-mass spectrometry (LC-MS) analysis (Shimadzu Corp., Kyoto, Japan, LCMS-2020). We used HC that contained a solution of echinochrome A sodium salts in ampoules. HC was used as a stock solution diluted with the appropriate solvent or culture medium.

### 2.2. Experimental Animals

The animal studies were approved by the Catholic University Institutional Animal Care and Use Committee and the Department of Laboratory Animals at the Catholic University of Korea.

### 2.3. Ischemia/Reperfusion Injury Model and In Vivo Drug Administration

We referred to the practical guideline for experimental models of myocardial ischemia and infarction [36]. Briefly, male Fischer 344 rats (8 weeks old and 160 to 180 g; KOATECH, Pyeongtaek-si, Korea) were anesthetized by inhalation with 2% isoflurane and intubated using an 18-gauge intravenous catheter. The rats were mechanically ventilated with medical-grade oxygen. Surgery was performed on a 37 °C heating pad to prevent the body from getting cold. A left thoracotomy was performed after the chest was shaved to prevent contamination during surgery.

Before ischemia, sterile polyethylene glycol tubing (22 gauge) was raised above the left anterior descending (LAD) artery and a suture was tied for 1 min for pre-conditioning. Then, a knot was made with 7–0 Prolene to achieve ischemic injury for 1 h. HC (1 mg/kg) was injected intravenously [26,32] 5 min before reperfusion. The chest was aseptically closed and disinfected after surgery. To establish the baseline LV function, EF and FS were examined 4 h after surgery.

### 2.4. Measurement of Myocardial Infarct Size

TTC and Evans blue staining was performed to determine the early cardiac protective effects of HC. The rats were anesthetized and ventilated as described previously. After 60 min of ischemia and 24 h of reperfusion, the suture thread around the LAD artery was retied and Evans blue dye (9% in PBS) was injected intravenously into the rats. After 15 min, the heart was quickly excised and incubated for 10 min at −4 °C. The heart was cut into 3 slices (about 2 mm thickness) and incubated with 2% TTC for 30 min at 37 °C in the dark. After washing three times, the tissue was fixed in 4% paraformaldehyde.

The non-infarcted myocardium was stained deep blue with Evans blue. The viable myocardium was stained red with TTC. The necrotic myocardium appeared white after TTC staining. The area at risk (AAR) and necrotic area were quantified using ImageJ software.

### 2.5. Evaluation of Heart Function by Echocardiography

Functional evaluation of the I/R-injured hearts was performed using echocardiography. The rats were lightly anesthetized with 2% isoflurane, and data were recorded using a transthoracic echocardiography system equipped with a 15 MHz L15-7io linear transducer (Philips, Amsterdam, Netherlands, Affniti 50G).

Serial echocardiograms were performed at baseline one, two, and four weeks after surgery. The echocardiography operator was blinded to the group allocation during the experiment. EF and FS, which are indexes of LV systolic function, were calculated with the following equations, respectively.

EF (%) = [(LVEDV-LVESV)/LVEDV] × 100/FS (%) = [(LVEDD-LVESD)/LVEDD] × 100

### 2.6. Hemodynamic Measurements

Hemodynamic measurements were performed before euthanasia at the endpoint of 4 weeks. The rats were lightly anesthetized with 2% isoflurane. Thoracotomy was performed without bleeding. Then, the LV apex of the heart was pierced with a 26-gauge needle and a 2F conductance catheter (Millar, Houston, TX, USA, SPR-838) was placed in the LV. The PV parameters were uninterruptedly recorded using a PV conductance system (emka TECHNOLOGIES, Paris, France, MPVS Ultra) coupled to a digital converter (ADInstruments, Dunedin, New Zealand, PowerLab 16/35). Load-independent measurements of cardiac function, including the slopes of ESPVR and EDPVR, were achieved with different preloads, which were elicited by IVC occlusion with a needle holder. Hypertonic saline (50 µL of 20% NaCl) was injected into the left jugular vein to evaluate the parallel conductance after hemodynamic measurements. Blood was collected from the LV into a heparinized syringe and placed into cuvettes to convert the conductance signal to volume using the catheter. The absolute volume of the rat was confirmed by calibrating the parallel conductance and the cuvette conductance.

### 2.7. Measurement of Capillary Density by Immunohistochemical Staining

The hearts were fixed in 4% paraformaldehyde overnight and paraffin blocks were made. The heart was cross-sectioned into 4 μm sections starting at the top of the apex using a microtome (Leica, Wetzlar, Germany, LRM2255). Immunofluorescence was performed to determine the capillary density of the injured hearts. After deparaffinization and rehydration, antigen retrieval with target retrieval solution was performed in a decloaking chamber. The sections were incubated with diluted primary antibody at 4 °C overnight. The primary antibodies used in this study were mouse anti-cardiac troponin t (1:200) (Abcam, Cambridge, UK, ab8295) and goat anti-CD31 (1:200) (R&D Systems, Minneapolis, MN, USA, AF3628). After washing four times with phosphate-buffered saline (PBS), the samples were incubated with secondary antibody for 90 min at room temperature in the dark. The secondary antibodies used in this study were donkey anti-mouse IgG (H+L) highly cross-adsorbed secondary antibody, Alexa Fluor 488 (1:500) (Invitrogen, Waltham, MA, USA, A-21202), and rabbit anti-goat IgG (H+L) cross-adsorbed secondary antibody, Alexa Fluor 594 (1:500) (Invitrogen, A-11080). After washing again with PBS, the sections were stained with an anti-fade mounting medium with 4′,6-diamidino-2-phenylindole (DAPI) (Vector Laboratories, Burlingame, CA, USA, H-1200-10) for nuclear staining and then mounted on slides.

The number of capillaries was counted in five random microscopic fields using a fluorescence microscope and expressed as the number of capillaries per square millimeter of tissue area.

### 2.8. Measurement of Myocardial Infarct Size by Masson’s Trichrome Staining

Masson’s trichrome staining (Sigma-Aldrich, Saint Louis, MO, USA, HT15) was performed to determine the fibrotic area, wall thickness scar area, and viable myocardium of the injured hearts. Paraffin slides were preincubated overnight in a 37 °C dry oven before deparaffinization and rehydration. After deparaffinization and rehydration, the paraffin sections were refixed for 1 h and 30 min in 56 °C Bouin’s solution. After that, the slides were washed with tap water for 20 min. The sections were stained using Weigert’s iron hematoxylin solution for 15 min at room temperature, followed by staining with Biebrich scarlet-acid fuchsin solution for 20 min at room temperature. Last, the sections were counterstained with aniline blue for 15 min and incubated in 1% acetic acid for 2 min at room temperature.

The collagen fibers appeared blue and the viable myocardium appeared red. Imaging of the heart sections was performed with a slide scanner (3DHISTECH Ltd, Budapest, Hungary, PANNORAMIC MIDI II). All other areas, including the fibrotic area, were quantified using ImageJ software.

### 2.9. Western Blotting

Heart tissues were homogenized in ice-cold T-PER™ Tissue Protein Extraction Reagent (Thermo Fisher Scientific, Waltham, MA, USA, 78510) with a complete Halt™ Protease and Phosphatase Inhibitor Cocktail (Thermo Scientific, 78440). Total protein was quantified by Pierce™ BCA Protein Assay Kit (Thermo Fisher Scientific, 23225). Take equal amounts of total protein sample and add a Fluorescent Compatible Sample Buffer (Invitrogen, LC2570) with 10X Bolt™ Sample Reducing Agent (Invitrogen, B0009). Next, boil each sample at 95 °C for 5 min and then centrifuge at 16,000× *g* for 1 min at 4 °C. The supernatant was collected for Western blotting.

Load equal amounts of protein were resolved by Bolt™ 4 to 12% Bis-Tris Protein Gel (Invitrogen, NW04122BOX) in each experiment. Next, proteins were transferred to iBlot™ 2 PVDF Transfer Stacks (Invitrogen, IB24002) and membranes were incubated with primary antibody overnight at 4 °C, and the secondary antibody for 1 h at room temperature.

For the quantification of target signal expression, all samples to be compared were run on the same gel and image. Total protein normalization was carried out using No-Stain™ Protein Labeling Reagent (Invitrogen, A44717) according to the assay kit’s instructions. Bands were quantified using iBright™ FL1500 Imaging System (Invitrogen, A44115).

The antibodies used in Western blotting were anti-Cox-2 (1:100) (Santa Cruz Biotechnology, Dallas, TX, USA, sc-376861), anti-GPx4 (1:100) (Santa Cruz Biotechnology, sc-166570), and Goat anti-Mouse IgG (H+L) Highly Cross-Adsorbed Alexa Fluor Plus 647 (1:5000) (Invitrogen, A32728).

### 2.10. Evaluation of Mitochondrial Damage Scores by Transmission Electron Microscopy

For transmission electron microscopic (TEM) observation, samples of the infarct zone (2 mm × 2 mm × 2 mm) were quickly removed from the LV and the specimens were fixed in 4% paraformaldehyde and 2.5% glutaraldehyde in 0.1 M phosphate buffer (PB) for 3 h. After rinsing in 0.1 M PB, the samples were post-fixed in 1% osmium tetroxide for 30 min. They were then dehydrated in a graded ethanol series (50, 70, 80, 90, 95, and 100%). The infiltrated samples were polymerized in Epon 812 at 60 °C overnight. Ultrathin sections were cut on an ultramicrotome (Leica, Ultracut UCT) to a thickness of about 60–70 nm. The sectioned slices were collected on grids (200 mesh) and stained with 2% uranyl acetate and lead citrate. The prepared grids were examined by transmission electron microscopy (JEOL, Tokyo, Japan, JEM 1010) at 60 kV.

The degree of ultrastructural damage was evaluated using Flameng scores [37]. Five fields of view were randomly selected in each sample and 20 mitochondria were evaluated in each field of view. According to the degree of mitochondrial damage, scores of 0–4 grades were made for each mitochondrion (the higher the level, the heavier the damage).

### 2.11. Neonatal Rat Cardiomyocyte Isolation

Neonatal rat cardiomyocytes (NRCMs) were isolated from 1-day-old Sprague-Dawley rat hearts following the guidelines and approval of the Animal Care and Use Committee at the Catholic University of Korea. After rapidly harvesting ventricle tissue and putting it in ice-cold Hanks’ balanced salt solution (HBSS) to remove blood, it was incubated with 0.1% trypsin solution (Welgene, Gyeongsan-si, Korea, LS 015-03) with gentle agitation at 4 °C overnight. The next day, the digested tissue was transferred to a Petri dish on ice, minced, and collected into a conical tube. Collagenase B (5 mL of 1 mg/mL) (Roche, Basel, Switzerland, 11088815001) in HBSS was added to the heart tissue, pipetted 10–15 times, and incubated in a 37 °C water bath for 5 min with gentle shaking. The supernatant containing the cells was transferred to fresh Dulbecco’s modified Eagle medium (DMEM) (Gibco, Amarillo, TX, USA, 12320032) containing 20% fetal bovine serum (FBS, Gibco, 10082147), and the undigested heart tissue was resuspended in 5 mL of collagenase B solution. The digestion step was repeated five times, and the cell-containing supernatant was collected. Non-cardiomyocyte populations were removed by Percoll density-gradient centrifugation for 30 min at 3000× *g* at 4 °C. After the Percoll density-gradient step, the purified NRCMs were plated at a density of 1 × 10^5^ per cm^2^ in gelatin-coated cultureware and cultured at 37 °C and 5% CO_2_. The culture medium contained 10% FBS and 1 × antibiotic-antimycotic solution (Gibco, 15240062).

### 2.12. Chemicals and Reagents

Hydrogen peroxide (H_2_O_2_) solution (216763), RSL3 (SML2234), erastin (E7781), menadione (M5625), rotenone (R8875), antimycin A (A8674), and ammonium iron (II) sulfate hexahydrate (09719) were purchased from Sigma-Aldrich. For the in vitro cell experiments, the compounds and HC were added to the assay medium simultaneously (DMEM supplemented with 1% FBS).

### 2.13. Cell Viability Assays

CCK-8 assay: Briefly, NRCMs at a density of 1 × 10^5^ per cm^2^ were seeded into a gelatin-coated 96-well culture plate. The cells were treated with vehicle (dimethyl sulfoxide (DMSO) or water), H_2_O_2_ (500 μM), menadione (80 μM), rotenone (20 μM), antimycin (20 μM), RSL3 (2 μM), erastin (40 μM), and HC for 2 h (oxidative stress induced) or 6 to 24 h (ferroptosis induction), and then changed to fresh assay medium. CCK-8 reagent (10 μL, Dojindo Molecular Technologies, Rockville, MD, USA, CK04) was added to each well of the plate and the plate was incubated for 1 h. The absorbance was measured at 450 nm using a microplate reader.

Live/Dead analysis: Cell viability was analyzed using the LIVE/DEAD Viability/Cytotoxicity Kit (Invitrogen, L3224) according to the manufacturer’s instructions. Briefly, after changing the medium to HBSS containing calcein (2 μM) and the ethidium homodimer-1 (4 μM) dye mixture, the NRCMs were incubated for 20 min at room temperature. Then, the labeled cells were analyzed with a fluorescence microscope. The viable cell area and number of dead cells were calculated by ImageJ software and normalized to the control group.

### 2.14. Quantitative Real-Time PCR with Reverse Transcription

Total RNA was extracted using TRIzol™ Reagent (Invitrogen, 15596018) following the general experimental protocol for RNA isolation. Complementary DNA was synthesized from 500 ng of total RNA using PrimeScript™ RT Master Mix (Perfect Real Time) (Takara, RR036A). Quantitative real-time PCR (qRT-PCR) was performed using TB Green^®^ Premix Ex Taq™ (Tli RNaseH Plus) (Takara Bio, Mountain View, CA, USA, RR420A) and the LightCycler^®^ 480 Instrument II (Roche). The mRNA transcript abundance was normalized to that of Actb and Hprt. The qRT-PCR primer sequences are described in Table S1.

### 2.15. Measurement of Cellular ROS and Mitochondrial Superoxide

General cellular ROS: Cellular ROS was measured using CellROX^®^ Oxidative Stress Reagents (Invitrogen, C10444). Briefly, NRCMs at a density of 1 × 10^5^ per cm^2^ were seeded in a gelatin-coated 96-well black polystyrene microplate (Corning^®^, Corning, NY, USA, CLS3603). The cells were treated with vehicle (DMSO), menadione, or menadione with HC for 1 h. The cells were stained with 5 μM CellROX^®^ reagent in the complete media and incubated at 37 °C for 30 min, then washed with HBSS and observed under a fluorescence microscope. Both the control and treated samples used the same exposure time. The fluorescence intensity was calculated by ImageJ software and normalized to the control group.

Mitochondrial superoxide: Mitochondrial superoxide was measured using the MitoSOX™ Red mitochondrial superoxide indicator (Invitrogen, M36008). Briefly, NRCMs at a density of 1 × 10^5^ per cm^2^ were seeded in a gelatin-coated 96-well black polystyrene microplate (Corning, CLS3603). The cells were treated with vehicle (DMSO), rotenone/antimycin A, or rotenone/antimycin A with HC for 1 h. The cells were stained with 5 μM MitoSOX™ reagent in HBSS and incubated at 37 °C for 10 min, then washed with HBSS and observed under a fluorescence microscope. Both the control and treated samples used the same exposure time. The fluorescence intensity was calculated by ImageJ software and normalized to the control group.

### 2.16. Measurement of Lipid Peroxidation

Lipid peroxidation was measured using a BODIPY™ 581/591 C11 (lipid peroxidation sensor) (Invitrogen, D3861). Briefly, NRCMs at a density of 1 × 10^5^ per cm^2^ were seeded in a gelatin-coated 96-well black polystyrene microplate (Corning, CLS3603). The cells were treated with vehicle (DMSO), RSL3, or RSL3 with HC for 90 min, and then washed once with HBSS. Next, the cells were incubated for 30 min with BODIPY™ 581/591 C11 (5 μM) in HBSS.

The fluorescence of BODIPY™ C11 was measured by the acquisition of green and red signals, providing a ratiometric indication of lipid peroxidation. Upon oxidation, the labeled BODIPY™ C11 fluorescence signal shifts from red (emission peak ~590 nm) to green (~510 nm). Both the control and treated samples used the same exposure time. The values were calculated by ImageJ software as the ratio of the green (oxidized probe) to red (reduced probe) fluorescence signal intensity and normalized to the control group.

### 2.17. Measurement of Glutathione Peroxidase Activity

Glutathione peroxidase activity was measured using a glutathione peroxidase assay kit (Cayman Chemical, Ann Arbor, MI, USA, 703102) according to the manufacturer’s instructions. Briefly, NRCMs at a density of 1 × 10^5^ per cm^2^ were seeded in a gelatin-coated 6-well culture plate. The cells were treated with vehicle (DMSO), RSL3, or RSL3 with HC for 2 h, and then washed once with PBS. Next, the cells were harvested using a cell lifter and the cells were homogenized in cold Tris-HCL buffer and then centrifuged at 10,000× *g* for 15 min at 4 °C. The supernatant was collected for analysis.

The analysis was performed according to the assay kit’s instructions, and the absorbance was read at a wavelength of 340 nm (A340) for 10 min at 1 min intervals. Glutathione peroxidase activity was calculated according to the assay kit’s formula.

Activity = (ΔA340 min/0.00373 μM^−1^) × (0.19 mL/0.02 mL) × Sample dilution = nmol/min/mL.

### 2.18. Measurement of Glutathione (GSH) and Oxidized Glutathione (GSSG)

Glutathione (GSH) and oxidized glutathione (GSSG) were measured using a glutathione colorimetric detection kit (Invitrogen™, EIAGSHC) according to the manufacturer’s instructions. Briefly, NRCMs at a density of 1 × 10^5^ per cm^2^ were seeded in a gelatin-coated 6-well culture plate. The cells were treated with vehicle (DMSO), erastin, or erastin with HC for 6 h, and then washed once with PBS. Next, the cells were harvested using a cell lifter and the cells were homogenized in ice-cold 5% 5-sulfosalicylic acid dihydrate (SSA, Sigma-Aldrich, S2130), centrifuged at 14,000 rpm for 10 min at 4 °C, and the supernatant was collected for analysis.

Heart tissues were homogenized in ice-cold phosphate buffer and then centrifuged at 14,000 rpm for 10 min at 4 °C. The supernatant was collected for analysis. Total protein was quantified by Pierce™ BCA Protein Assay Kit (Thermo Fisher Scientific, 23225).

The samples were treated with 2 VP (2-vinylpyridine) (Sigma-Aldrich, 132292) to measure GSSG. Analysis was performed according to the assay kit’s instructions, and absorbance was read at a wavelength of 405 nm (A405). Glutathione concentrations were calculated from the standard curve.

### 2.19. Detection of Intracellular Ferrous Ions (Fe^2+^)

Intracellular iron and the chelating effect of HC were measured using FerroOrange (Dojindo Molecular Technologies, F374). Briefly, NRCMs at a density of 1 × 10^5^ per cm^2^ were seeded in a gelatin-coated 96-well black polystyrene microplate (Corning, CLS3603). The cells were treated with vehicle (water), ammonium iron (II) sulfate (200 μM), or ammonium iron (II) sulfate with HC for 1 h in serum-free media, and then washed with HBSS three times. Next, FerroOrange (1 μM) was added to the cells in HBSS and the cells were incubated for 30 min. Then, the cells were observed under a fluorescence microscope without a wash step.

Both the control and treated samples used the same exposure time. Fluorescence intensity was calculated by ImageJ software and normalized to the control group.

### 2.20. Statistical Analysis

All data were presented as the mean ± SEM using Image GraphPad Prism 9.2 software (GraphPad Software, San Diego, CA, USA) unless otherwise indicated. The groups were compared using either a two-tailed unpaired *t*-test or ANOVA, when appropriate. The statistical differences among three or more groups were also analyzed by one- or two-way ANOVA followed by Tukey’s test. Differences with *p*-values of less than 0.05 were considered significant.

## 3. Results

### 3.1. Cardioprotective Effects of Histochrome on Myocardial I/R Injury

**Figure 1:**
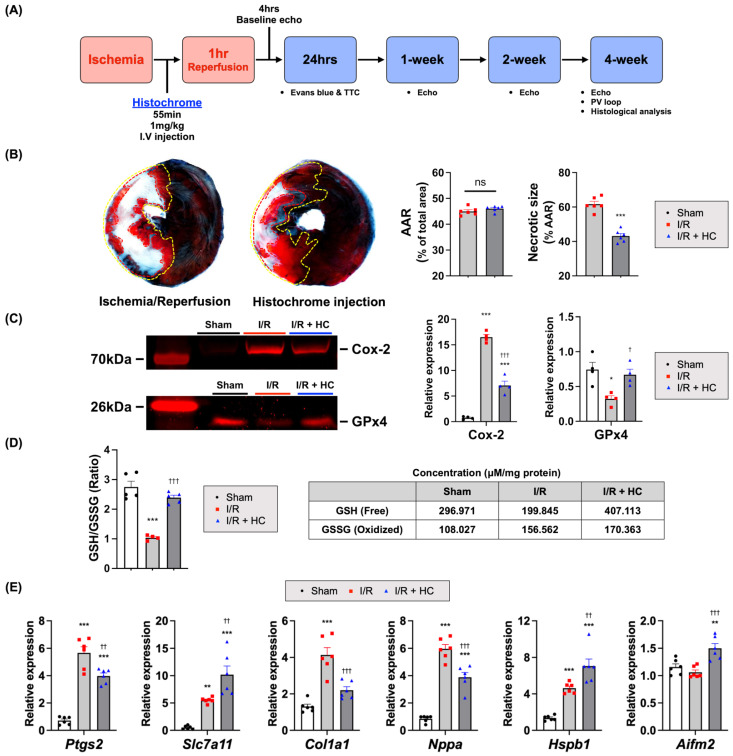
Cardioprotective effects of HC against myocardial I/R injury. (**A**) Experimental process. HC (1 mg/kg) was injected intravenously 5 min before reperfusion. (**B**) The representative images of TTC/Evans blue staining 24 h after reperfusion. The area at risk (AAR) was not significantly different between the I/R control and the HC group, but necrotic size (white) was significantly reduced in the HC injection group. ^ns^ *p* = 0.19; *** *p* < 0.001 versus I/R (*n* = 6/group) by unpaired *t*-test. Error bars show means ± SEM. (**C**) The relative images of Western blot analysis (anti-Cox-2, anti-GPx4) 3 days after I/R injury. The relative signals were calculated and are shown after normalizing to total protein (Figure S1B). * *p* < 0.05; *** *p* < 0.001 versus Sham; † *p* < 0.05; ††† *p* < 0.001 versus I/R (*n* = 4/group) by one-way ANOVA. Error bars show means ± SEM. (**D**) The ratio of glutathione (GSH):oxidized glutathione (GSSG) 3 days after I/R-injured heart tissues. *** *p* < 0.001 versus Sham; ††† *p* < 0.001 versus I/R (*n* = 4 or 5/group) by one-way ANOVA. Error bars show means ± SEM. (**E**) The relative mRNA expression level of ferroptosis and heart failure-related genes using heart tissue harvested 3 days after I/R injury. ** *p* < 0.01; *** *p* < 0.001 versus Sham; †† *p* < 0.01; ††† *p* < 0.001 versus I/R (*n* = 6/group) by one-way ANOVA. Error bars show means ± SEM.

Given the significant protective effects of HC against ischemic injury in the heart reported in several previous studies, we first sought to reconfirm the therapeutic effects of HC on myocardial I/R injury in our setting [32,33,34]. For this purpose, we administered HC (1 mg/kg) intravenously 60 min after ischemic injury and 5 min before reperfusion (Figure 1A). The infarct size (IS) and area at risk (AAR) of each rat were evaluated by 2,3,5-Triphenyltetrazolium chloride (TTC)/Evans blue staining 24 h after reperfusion (Figure 1B). Although the AAR of the myocardium was similar between the groups, HC-treated rat hearts exhibited significantly smaller infarct size compared to the control group, implying an early cardioprotective effect of HC on I/R injury.

Next, to address whether the cardioprotection was associated with the anti-ferroptotic effect of HC, we evaluated the expression level of several genes and proteins related to ferroptosis using heart tissues harvested three days after I/R injury (Figure S1A). The Western blotting data showed that glutathione peroxidase 4 (GPx4) protein expression level significantly decreased, and cyclooxygenase-2 (Cox-2) protein was evident after I/R injury (Figure 1C). The qRT-PCR data also showed increased *Ptgs2* and *Chac1* gene expression levels (Figure 1E and Figure S1C). GPx4 is a selenocysteine-containing enzyme considered to be a master regulator to prevent ferroptosis [38,39,40,41]. The decreased expression level of GPx4 during ischemic injury contributes to ferroptotic cell death [42] and Cox-2 is considered for biomarkers of ferroptosis [39,43,44].

Interestingly, HC treatment not only decreased *Ptgs2* and *Chac1* gene expression and Cox-2 protein expression levels, but also increased *Slc7a11*, *Aifm2*, and *Hspb1* gene expression and GPx4 protein expression levels (Figure 1C,E and Figure S1C). *Slc7a11* is involved in the production of glutathione (GSH), an important cellular antioxidant, and *Aifm2* is known as a negative regulator of ferroptosis, which had a protective effect on the cooperation with GPx4 to suppress the lipid peroxidation and ferroptosis processes [45,46]. Upregulation of heat-shock protein beta 1 (HSPB1) decreases intracellular iron and therefore increases *Hspb1* expression significantly, inhibiting ferroptotic cell death [47,48].

Upon MI, GSH was depleted in cardiomyocytes and gradually increased oxidative stress, leading to accumulation of glutathione disulphide (GSSG), an oxidized form of GSH [49]. A recent report showed that pathological conditions such as MI-induced heart failure cause intracellular GSH depletion [50] and lead to ferroptosis [17,42]. We also confirmed that the GSH level was decreased after I/R injury, and HC treatment significantly increased free GSH and the ratio of GSH (reduced):GSSG (oxidized) compared to the control group (Figure 1D).

In addition, HC treatment significantly decreased *Nppa*, *Nppb*, *Col1a1*, and *Col3a1* levels, known as major heart failure markers (Figure 1E and Figure S1C). These findings suggest that the cardioprotective effect of HC against I/R injury might be associated with anti-ferroptotic cell death.

### 3.2. Prolonged Cardioprotective Effects of Histochrome on Myocardial I/R Injury

**Figure 2:**
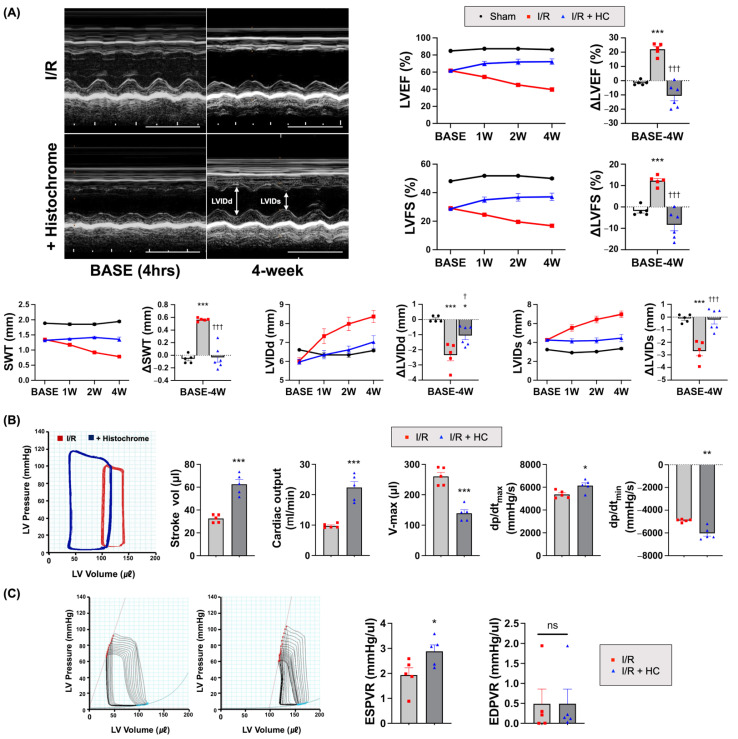
Prolonged cardioprotective effects of HC on myocardial I/R injury. (**A**) The representative M-mode echocardiography images at base line (4 h) and 4 weeks after HC treatment. Scale bar = 0.5 s. Longitudinal assessment of heart function by echocardiography (echocardiographic parameters in Figure S2). Differences in left ventricular ejection fraction (EF), fractional shortening (FS), septal wall thickness (SWT), end-diastolic (LVIDd) and -systolic dimensions (LVIDs) between baseline and 4 weeks after HC injection. * *p* < 0.05; *** *p* < 0.001 versus Sham; † *p* < 0.05; ††† *p* < 0.001 versus I/R by one-way ANOVA. Error bars show means ± SEM. (**B**) The representative images of hemodynamic pressure and volume (PV) loop at 4 weeks. Stroke volume, cardiac output, and volume max (V-max) defining the amount of blood volume in the left ventricular at the end diastole. The maximum rate of pressure change (dP/dt_max_) during systole and minimal rate of pressure changes (dP/dt_min_) during diastole. * *p* < 0.05; ** *p* < 0.01; *** *p* < 0.001 versus I/R (*n* = 5/group) by unpaired *t*-test. Error bars show means ± SEM. (**C**) Load-independent measures of cardiac contractility via temporary occlusion of the inferior vena cava (IVC). Slope of end-systolic pressure–volume relationship (ESPVR) indicating the intrinsic cardiac contractility. Slope of end-diastolic pressure–volume relationship (EDPVR). ^ns^ *p* > 0.99; * *p* < 0.05 versus I/R control (*n* = 5/group) by unpaired *t*-test. Error bars show means ± SEM.

To evaluate whether the cardioprotective effects from a single intravenous treatment with HC could be prolonged over the long term, we performed serial echocardiography at baseline one, two, and four weeks after HC treatment (Figure 2A). The LV ejection fraction (EF) and fractional shortening (FS) were significantly higher in the HC treatment group than in the I/R control group. In addition, HC treatment preserved LV septal wall thickness (SWT) as well as end-diastolic (LVIDd) and -systolic dimensions (LVIDs) better than the I/R control. These findings indicate that a single and acute HC treatment could provide long-term benefits in terms of cardiac function and remodeling in I/R-injured hearts.

Since the cardiac function measured by echocardiography is load-dependent, we further performed pressure–volume (PV) loop studies to determine the intrinsic cardiac contractibility four weeks post-MI. Several parameters related to cardiac contractility, such as cardiac output (CO), stroke volume (SV), the maximum rate of pressure change (dP/dt_max_), and the minimum rate of pressure change (dP/dt_min_), were significantly higher in the HC treatment group than in the I/R control group (Figure 2B). Moreover, as a cardiac remodeling index, the volume max (V-max) was lower in the HC treatment group than in the I/R control group, indicating that HC decreased adverse cardiac remodeling after I/R injury.

We also measured load-independent cardiac contractility via temporary occlusion of the inferior vena cava (IVC). The HC treatment exhibited a significantly steeper slope of the end-systolic pressure–volume relationship (ESPVR) than the I/R control group. The slope of the end-diastolic pressure–volume relationship (EDPVR) was comparable between the groups (Figure 2B,C). The results from the PV loop analysis verified that HC treatment led to a significant hemodynamic improvement in cardiac contractibility in I/R-injured hearts.

### 3.3. Histochrome Ameliorates Effect in Infarcted Hearts by Reducing Adverse Cardiac Remodeling after I/R

**Figure 3:**
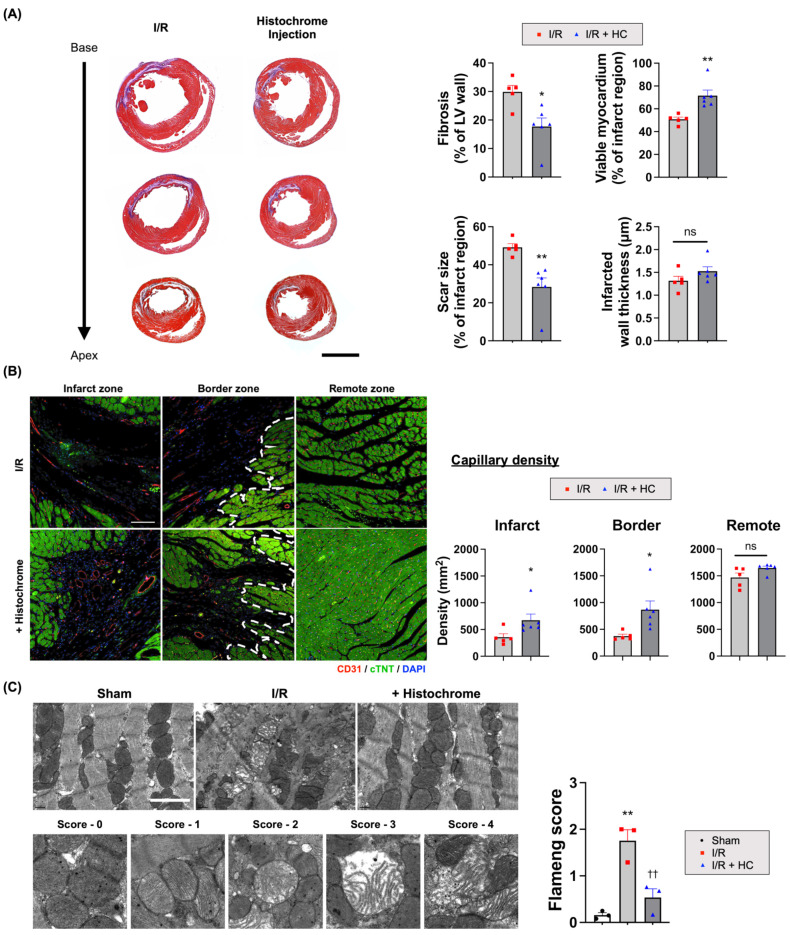
The therapeutic effects of HC on CM protection and fibrosis. (**A**) The representative images of Masson’s trichrome (MT) staining using the heart tissues harvested at 4 weeks after I/R injury. Purple and red indicate fibrotic tissue and viable myocardium, respectively. Quantification summary of a percentage of fibrosis, scar size, viable myocardium, and thickness. ^ns^ *p* = 0.16; * *p* < 0.05; ** *p* < 0.01 versus I/R (*n* = 5 or 6/group) by unpaired *t*-test. Error bars show means ± SEM. Scale bar = 4000 μm. (**B**) The representative images of capillary density stained with anti-CD31 (red) and anti-cTNT (green) on the infarct, border, and remote zone at 4 weeks after I/R injury. For quantification, the number of capillaries on five randomly selected fields from three different levels were counted. ^ns^ *p* = 0.06; * *p* < 0.05 versus I/R (*n* = 5 or 6/group) by unpaired *t*-test. Error bars show means ± SEM. Scale bar = 200 μm. (**C**) The representative transmission electron microscopic (TEM) images of heart tissue at 72 h after reperfusion. The degree of ultrastructural damage was evaluated using Flameng scores (Figure S7B). ** *p* < 0.01 versus Shame; †† *p* < 0.01 versus I/R (*n* = 3/group) by one-way ANOVA. Error bars show means ± SEM. Scale bar = 2 μm.

In infarcted hearts, CM loss and fibroblast infiltration trigger fibrosis by secreting collagen and other matrix proteins, which deteriorate cardiac function and promote adverse remodeling [51,52,53]. To verify the therapeutic effects of HC treatment on CM protection and fibrosis, we performed Masson’s trichrome staining of the post-MI hearts at four weeks, which showed that the HC treatment increased viable myocardium in the infarct region and reduced the percentage of LV wall fibrosis compared to the I/R controls (Figure 3A and Figure S6).

We also measured mitochondrial damage scores in the infarcted heart tissue using transmission electron microscopy. I/R injury disarranged mitochondrial structure in the CMs and reduced cristae density. However, HC treatment significantly reduced the mitochondrial damage (Flameng scores) compared to those of the I/R controls (Figure 3C and Figure S7B). Furthermore, we also performed CD31 immunohistochemical staining to assess the capillary density, which also showed increased capillary density in both the border and infarct zone of the injured hearts of the HC treatment group compared to the I/R control group (Figure 3B).

### 3.4. Histochrome Protects the Cardiomyocytes from I/R Injury via an Antioxidant Effect

**Figure 4:**
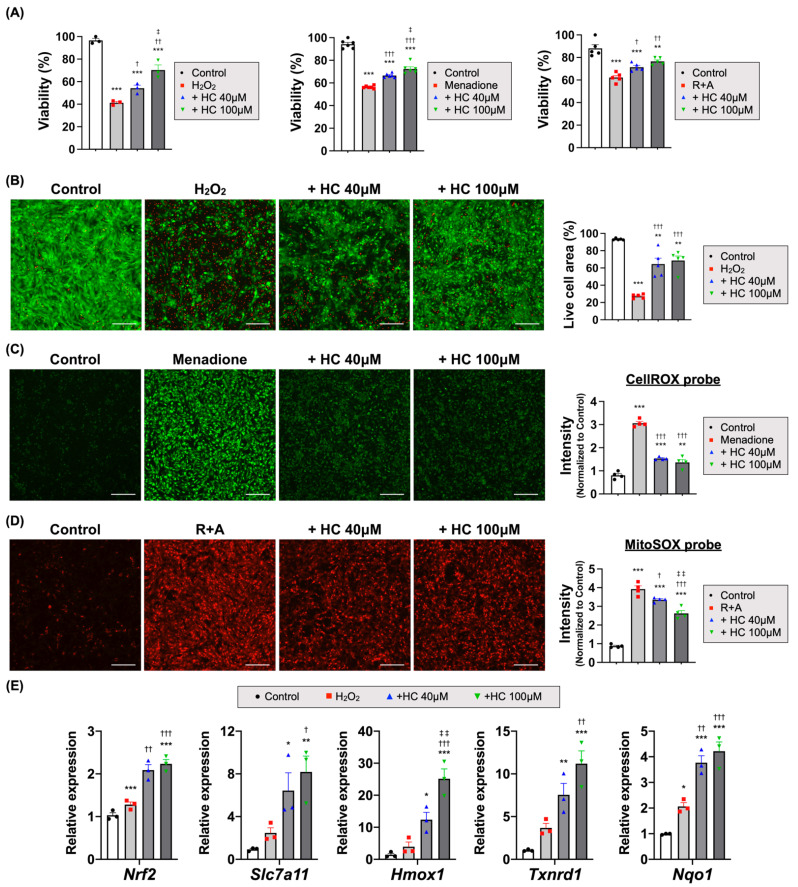
HC protected CMs under oxidative stress via an antioxidant effect. (**A**) Viability of NRCMs treated with various types of oxidative stress-inducing drugs (2 h). *** *p* < 0.001 versus Control; † *p* < 0.05; †† *p* < 0.01; versus H_2_O_2_; ‡ *p* < 0.01 versus HC + 40 μM (n = 3/group, left). *** *p* < 0.001 versus Control; ††† *p* < 0.001 versus Menadione; ‡ *p* < 0.01 versus HC + 40 μM (n = 6/group, middle). ** *p* < 0.01; *** *p* < 0.001 versus Control; † *p* < 0.05; †† *p* < 0.01; versus or rotenone/antimycin A (R+A) (n = 5/group, right) by one-way ANOVA. Error bars show means ± SEM. (**B**) The representative images of Live/Dead analysis treated with H_2_O_2_ and quantitative analysis of live cell area (green fluorescence). ** *p* < 0.01; *** *p* < 0.001 versus Control; ††† *p* < 0.001 versus H_2_O_2_, (*n* = 5/group) by one-way ANOVA. Error bars show means ± SEM. Scale bar = 200 μm. (**C**) The representative images of cellular ROS treated with menadione and quantitative analysis of probe intensity (CellROX, green fluorescence). ** *p* < 0.01; *** *p* < 0.001 versus Control; ††† *p* < 0.001 versus Menadione (*n* = 4/group) by one-way ANOVA. Error bars show means ± SEM. Scale bar = 200 μm. (**D**) The representative images of mitochondrial superoxide treated with rotenone/antimycin A (R+A) and quantitative analysis of probe intensity (MitoSOX, red fluorescence). *** *p* < 0.001 versus Control; † *p* < 0.05; ††† *p* < 0.001 versus R+A, ‡‡ *p* < 0.01 versus + HC 40 μM (*n* = 4/group) by one-way ANOVA. Error bars show means ± SEM. Scale bar = 200 μm. (**E**) The relative mRNA expression level of antioxidant-related genes in NRCMs treated with H_2_O_2_. * *p* < 0.05; ** *p* < 0.01; *** *p* < 0.001 versus Control; † *p* < 0.05; †† *p* < 0.01; ††† *p* < 0.001 versus H_2_O_2_; ‡‡ *p* < 0.01 versus HC + 40 μM (*n* = 3/group) by one-way ANOVA. Error bars show means ± SEM.

Given that significant anti-oxidant effects of HC were reported in several previous studies, to identify the underlying antioxidant mechanisms and modes of action of HC, we performed experiments to evaluate the viability of neonatal rat cardiomyocytes (NRCMs) after exposing them to various types of oxidative stress conditions induced by hydrogen peroxide, futile redox cycling (Menadione) [54,55], or mitochondrial complex I (rotenone) and III inhibitors (antimycin A) in the presence or absence of HC [56,57]. HC treatment significantly increased NRCM viability in all test groups in a dose-dependent manner compared to NRCMs under basal oxidative stress conditions (Figure 4A). Live/Dead analysis also showed that the viable cell area was higher in the HC-treated groups than in the H_2_O_2_ controls (Figure 4B and Figure S2A).

Next, we compared the antioxidant-related gene expression pattern of NRCMs between the HC-treated groups and the H_2_O_2_ controls. Interestingly, HC treatment increased *Nrf2*, *Slc7a11*, *Hmox1*, *Txnrd1*, and *Nqo1* (Figure 4E and Figure S2B), which control the basal and induced expression of antioxidant genes to oxidant exposure [58]. In addition, HC treatment significantly reduced the intracellular ROS levels induced by menadione (Figure 4C), and the mitochondrial superoxide levels induced by rotenone and antimycin A (Figure 4D). These findings suggest that HC protected CMs under oxidative stress via an antioxidant effect through *Nrf2* and its downstream pathway.

### 3.5. Histochrome Protected NRCMs from RSL3 and Erastin-Induced Ferroptotic Cell Death

**Figure 5:**
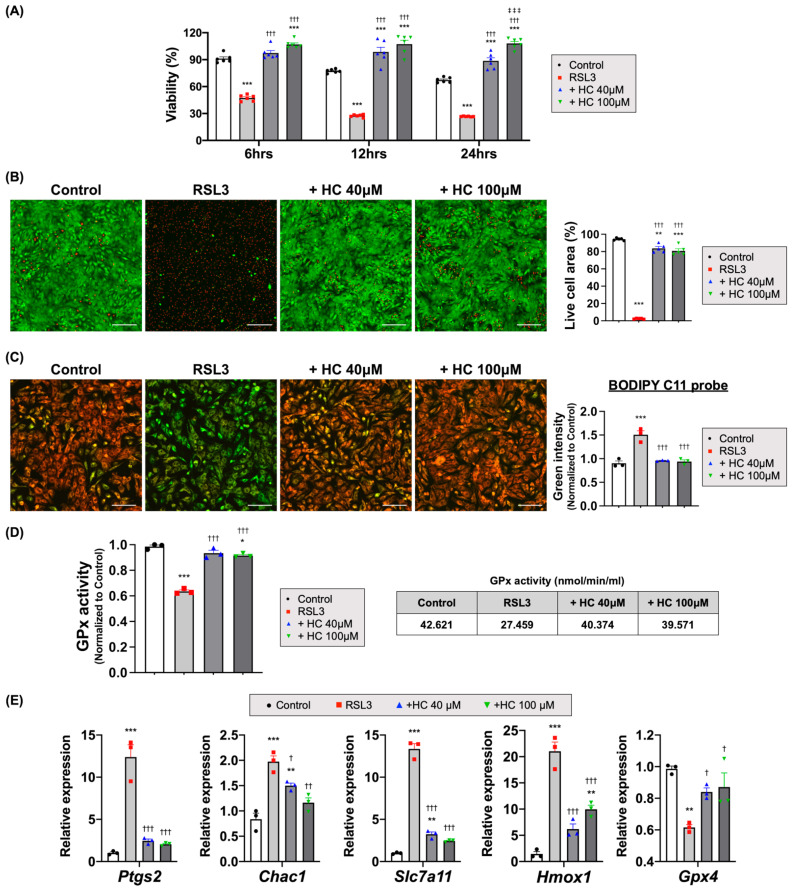
HC protected CMs from RSL3-induced ferroptotic cell death. (**A**) Viability of NRCMs treated with RSL3 at various time points. *** *p* < 0.001 versus Control; ††† *p* < 0.001 versus RSL3; ‡‡‡ *p* < 0.001 versus + HC 40 μM (*n* = 6/group) by one-way ANOVA. Error bars show means ± SEM. (**B**) The representative images of Live/Dead analysis treated with RSL3 and quantitative analysis of live cell area (green fluorescence). ** *p* < 0.01; *** *p* < 0.001 versus Control; ††† *p* < 0.001 versus RSL3 (*n* = 5/group) by one-way ANOVA. Error bars show means ± SEM. Scale bar = 200 μm. (**C**) The representative images of lipid peroxidation sensor (BODIPY 581/591 C11) and quantitative analysis of oxidized probe signal. Upon oxidation, labeled fluorescence signal shifts from red to green. *** *p* < 0.001 versus Control; ††† *p* < 0.001 versus RSL3 (*n* = 3/group) by one-way ANOVA. Error bars show means ± SEM. Scale bar = 200 μm. (**D**) GPx activity of NRCMs treated with RSL3 for 2 h. Activity was calculated according to the assay kit’s formula. * *p* < 0.05; *** *p* < 0.001 versus Control; ††† *p* < 0.001 versus RSL3 (*n* = 3/group). Error bars show means ± SEM. (**E**) The relative mRNA expression level of ferroptosis-related genes in NRCMs treated with RSL3. ** *p* < 0.01; *** *p* < 0.001 versus Control; † *p* < 0.05; †† *p* < 0.01; ††† *p* < 0.001 versus RSL3 (*n* = 3/group) by one-way ANOVA. Error bars show means ± SEM.

**Figure 6:**
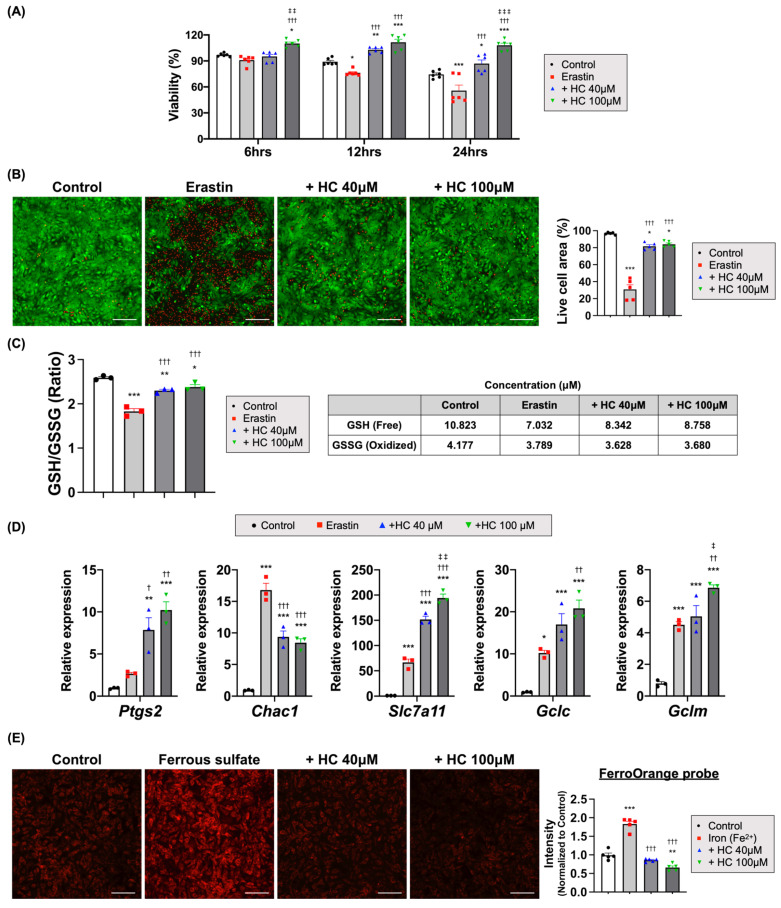
HC protected CMs from erastin-induced ferroptotic cell death. (**A**) Viability of NRCMs treated with erastin at various time points. * *p* < 0.05; ** *p* < 0.01; *** *p* < 0.001 versus Control; ††† *p* < 0.001 versus Erastin; ‡‡ *p* < 0.01; ‡‡‡ *p* < 0.001 versus + HC 40 μM (*n* = 6/group) by one-way ANOVA. Error bars show means ± SEM. (**B**) The representative images of Live/Dead analysis treated with erastin and quantitative analysis of live cell area (green fluorescence). * *p* < 0.05; *** *p* < 0.001 versus Control; ††† *p* < 0.001 versus Erastin (*n* = 5/group) by one-way ANOVA. Error bars show means ± SEM. Scale bar = 200 μm. (**C**) The ratio of glutathione (GSH):oxidized glutathione (GSSG) in NRCMs treated with erastin for 6 h. * *p* < 0.05; ** *p* < 0.01; *** *p* < 0.001 versus Control; ††† *p* < 0.001 versus Erastin (*n* = 3/group) by one-way ANOVA. Error bars show means ± SEM. (**D**) The relative mRNA expression level of ferroptosis-related genes in NRCMs treated with erastin. * *p* < 0.05; ** *p* < 0.01; *** *p* < 0.001 versus Control; † *p* < 0.05; †† *p* < 0.01; ††† *p* < 0.001 versus Erastin; ‡ *p* < 0.05; ‡‡ *p* < 0.01 versus HC + 40 μM (*n* = 3/group) by one-way ANOVA. Error bars show means ± SEM. (**E**) The representative images of intracellular iron treated with ammonium iron (II) sulfate and quantitative analysis of probe intensity (FerroOrange, red fluorescence). ** *p* < 0.01; *** *p* < 0.001 versus Control; ††† *p* < 0.001 versus Iron (Fe^2+^) (*n* = 5/group) by one-way ANOVA. Error bars show means ± SEM. Scale bar = 200 μm.

To investigate whether HC treatment protected the CMs from ferroptosis, an iron-mediated form of cell death provoked by the accumulation of lipid peroxidation products in the cell membrane during I/R injury [17,59], we first confirmed ferroptotic cell death via ferroptosis inducers such as RSL3 and erastin. RSL3 is a class 2 inducer that suppresses GPx4 directly, and erastin is a class 1 inducer that suppresses cystine/glutamate transporter (xCT) [39,60]. We observed that the treatment of cultured NRCMs with RSL3 induced rapid cell death and decreased cell viability to less than ~50% after 6 h of treatment (Figure 5A). However, HC treatment significantly augmented the cell viability, as evidenced by both CCK and Live/Dead analyses compared to the RSL3-treated control group (Figure 5B and Figure S3A,B). Treatment with erastin also showed similar results, that the cell viability began to decrease significantly starting after 12 h of erastin treatment and was less than ~60% by 24 h. However, there was no decrease in cell viability (Figure 6A) and higher viable cell areas were seen in the HC-treated group (Figure 6B and Figure S4A,B).

RSL3 treatment increased the mRNA expression level of *Ptgs2* as a ferroptosis marker [61,62], together with those of *Chac1*, *Slc7a11*, and *Hmox1*, and decreased *Gpx4*. However, HC treatment reversed the expression levels of *Ptgs2*, *Chac1*, *Slc7a11*, and *Hmox1* and increased *Gpx4* compared to those of RSL3-treated cells (Figure 5E and Figure S3C). In contrast, erastin treatment showed different gene expression patterns compared to those of RSL3-treated NRCMs. Erastin treatment also increased the mRNA expression level of *Ptgs2* together with those of *Chac1*, *Slc7a11*, and *Hmox1* but did not decrease *Gpx4*. HC treatment did not reverse the expression levels of *Ptgs2*, *Slc7a11*, and *Hmox1* compared to those of erastin treatment (Figure 6D and Figure S4C).

### 3.6. Histochrome Attenuates Lipid Peroxidation by Upregulating Glutathione Peroxidase Activity and Iron-Chelating Capacity

In previous experiments, we demonstrated that HC could significantly inhibit cell death by the ferroptosis inducer, RSL3. It directly acts on GPx4 and inhibits its activity. GPx4 has a fundamental role in ferroptosis by inhibiting the formation of lipid peroxides [47,63,64]. GPx4 converts GSH into oxidized GSSG and reduces toxic lipid peroxides (L-OOH) to non-toxic lipid alcohols (L-OH) [47,65]. Therefore, we investigated whether HC treatment could reduce ferroptosis-associated lipid peroxidation and restore antioxidant-related GPx4 activity after treatment with RSL3. Interestingly, whereas RSL3 treatment increased the BODIPY C11 sensor intensity of the oxidized probe and decreased GPx4 activity compared to the controls, HC treatment reversed the intensity of the oxidized probe and restored the GPx4 activity to a level similar to that of the control group (Figure 5C,D and Figure S5).

System xCT is a cystine-glutamate antiporter that is widely distributed in phospholipid bilayers and composed of two subunits (SLC7A11 and SLC3A2). Because GSH is generally synthesized from cysteine, xCT is an important antioxidant system in cells [66,67,68]. Therefore, the inhibition of xCT results in the depletion of GSH, thereby triggering ferroptosis. In this study, erastin treatment decreased GSH production by inhibiting xCT, but HC treatment increased the amount of free GSH and the GSH/GSSG ratio similar to the levels in the control group (Figure 6C). The mRNA expression levels of *Slc7a11*, *Gclc*, and *Gclm*, which are involved in the metabolism of GSH, were also significantly increased in the HC-treated groups (Figure 6D and Figure S4C). A previous study showed that increased transcription levels of *Slc7a11*, *Gclc*, and *Gclm* protected cells from erastin-induced ferroptosis and increased intracellular GSH [69,70]. Our results also showed that the increase in mRNA expression levels of *Slc7a11*, *Gclc*, and *Gclm* correlated with the increases in intracellular GSH and the maintenance of cellular homeostasis.

Iron is the most abundant and important trace element in the body, and it participates in various physiological processes [71]. Intracellular ferrous ion (Fe^2+^) accumulation can oxidize lipids and cause oxidative stress via a Fenton-like reaction, which promotes ferroptosis [17,59]. To assess the iron-chelating activity of HC, we measured the intracellular iron levels using a FerroOrange probe after treatment with ammonium iron (II) sulfate. HC treatment chelated the intracellular iron, which significantly reduced the signal intensity of intracellular Fe^2+^ in the NRCMs (Figure 6E). Therefore, these findings suggest that HC treatment could prevent ferroptotic cell death via iron chelation.

## 4. Discussion

Although the establishment of early and successful reperfusion is the most effective therapeutic strategy for improving clinical outcomes following acute myocardial ischemia, paradoxically, the restoration of blood flow and reperfusion to the ischemic myocardium after a period of ischemia allows for more damage to occur [5,6]. This irreversible injury has been termed myocardial I/R injury and accounts for up to 50% of the final size of the myocardial infarction [72]. Numerous subsequent studies ultimately identified that I/R injury is a complex pathological cellular event and involves a number of processes, including the generation of ROS and intracellular Ca^2+^ overload, all of which interact to mediate myocardial injury [2,73,74]. Although caspase-dependent apoptosis has long been recognized as the primary form of cell death in the heart [10,11,16], several recent studies reported that I/R injury is also aggravated by other regulatory mechanisms and signaling pathways such as elevated lipid peroxidation and intracellular iron levels [9,11,59].

In the present study, we demonstrated that the intravenous administration of HC prior to the reperfusion period following myocardial ischemia significantly protected the myocardium from I/R injury, attenuated adverse cardiac remodeling, and prevented cardiac fibrosis, ultimately leading to the improvement of cardiac function. Through a series of in vitro experiments to identify the detailed underlying molecular mechanisms of HC against myocardial I/R injury, we confirmed that HC treatment increased GPx4 protein expression and free GSH level after I/R-injured heart tissue, which are considered two major key regulators of ferroptosis.

Recently, many studies have focused on the discovery of new strategies to increase the expression of these factors in various pathological conditions [69,70,75,76,77]. We also provide proof of causality between the inhibition of ferroptosis by HC treatment and the improvement of adverse remodeling and cardiac function. First, we observed that the treatment of NRCMs with HC substantially reduced the levels of total intracellular ROS and mitochondrial superoxide accompanied by the significantly enhanced expression of *Nrf2*, a master regulator of redox homeostasis, as well as several other antioxidant-related genes such as *Slc7a11* and *Txnrd1* [58,78,79]. In subsequent analyses, we found that HC directly inhibited RSL3 or erastin-induced ferroptotic cell death in NRCMs by treatment with the ferroptosis inducer RSL3 or erastin, possibly by maintaining intracellular GSH levels as well as upregulating the activity of GPx4. In addition, HC directly lowered the expression level of several ferroptosis marker genes such as *Ptgs2*, *Chac1*, *Slc7a11*, and *Hmox1*, which were upregulated in the ferroptosis-induced NRCMs [61,62,80,81]. Lastly, in the experiments to test whether HC treatment could reduce ferroptosis-associated lipid peroxidation and restore GPx4 activity in ferroptosis-induced NRCMs, we demonstrated that whereas the RSL3 treatment augmented the intensity of the oxidized probe and decreased GPx4 activity compared to the control, HC treatment reversed the intensity of the oxidized probe and restored GPx4 activity similar to the levels in the control group.

Taken together, early intervention with HC prior to reperfusion can safely rescue the myocardium from I/R injury by preventing ferroptosis-induced cell death via its powerful antioxidant [27,28] and iron-chelating activities [29,30,31]. To the best of our knowledge, our study is the first to report that the cardioprotective effect of HC against myocardial I/R injury is through preventing ferroptotic cell death. Several clinical studies indicate that residual myocardial iron is one of the major risk factors for adverse left ventricular remodeling following I/R [82,83] and that iron chelation can successfully ameliorate myocardial I/R injury and subsequent heart failure [84,85]. Thus, drugs targeting ferroptosis can serve as an effective potential strategy to prevent ischemic cardiomyopathy.

HC is the biologically active secondary metabolite of sea urchins (Echinoidea) [25] and has been used widely in the Asian Pacific region for a significant time due to its high antioxidant activity [27,28]. As a water-soluble natural marine substance [26], HC is already used in daily clinical practice as an antioxidant drug without serious complications [26,34,86]. No study has reported that HC treatment modified the functional parameters in the liver and kidney; caused alterations in blood indexes, particularly in hemoglobin and hematocrit levels; or caused any allergic reactions. HC has also been prescribed for treating corneal diseases to protect from dystrophic damages of the retina and diabetic retinopathy [86].

There are some limitations when interpreting the results of this study. First, I/R injury can cause tissue damage not only in CMs but also in various resident immune cells and fibroblast and endothelial cells [7,87]. However, our in vitro experiments were performed only targeting CMs and associated with molecular biological changes. Second, we tried to focus on the ferroptosis regulatory effect of HC. Although we confirmed the direct anti-ferroptotic effects of HC by using well-known ferroptotic drugs such as RSL3 and erastin, we could not evaluate the role of HC in other forms of cell death such as apoptosis, necroptosis, and pyroptosis. Third, the sample size was relatively small, particularly in the in vitro experiment, even if it was statistically significant.

## 5. Conclusions

I/R injury not only upregulated Cox-2, but also downregulated GPx4 expressions. HC treatment significantly increased GPx4 and free GSH levels, but decreased Cox-2 level. HC treatment significantly decreased intracellular and mitochondrial ROS levels by upregulating the expression of *Nrf2* and antioxidant genes. HC also inhibited erastin- and RSL3-induced ferroptosis in CMs by maintaining intracellular GSH levels and upregulated the activity of glutathione peroxidase.

Our results demonstrated the substantial cardioprotective effects of HC against myocardia I/R injury by reducing ferroptosis-associated myocardial injury. As such, this study carries significant implications for providing secondary cardioprotection for AMI patients treated with primary PCI as a promising therapeutic option.

## Data Availability

The data presented in this study are available in article and supplementary material.

## Supplementary Materials

The following are available online at https://www.mdpi.com/article/10.3390/antiox10101624/s1. Figure S1: The relative mRNA expression level of ferroptosis-related genes after I/R injury. Figure S2: Echocardiographic parameters. Figure S3: NRCMs treated with H_2_O_2_, associated with Figure 4. Figure S4: NRCMs treated with RSL3, associated with Figure 5. Figure S5: NRCMs treated with erastin, associated with Figure 6. Figure S6: Measurement of lipid peroxidation and GPx activity. Figure S7: The representative images of MT staining and evaluation of mitochondrial damage. Table S1: Primers used for real-time PCR analysis.
