# Supplementary material for: Histochrome Attenuates Myocardial Ischemia-Reperfusion Injury by Inhibiting Ferroptosis-Induced Cardiomyocyte Death
Source: Antioxidants (Basel). 2021 Oct 15;10(10):1624. doi: 10.3390/antiox10101624 (PMC8533175; doi:10.3390/antiox10101624)
Supplement: Supplementary file 1 [file antioxidants-10-01624-s001.zip › antioxidants-1426903-supplementary.pdf]

antioxidants

MDPI

Supplementary figures

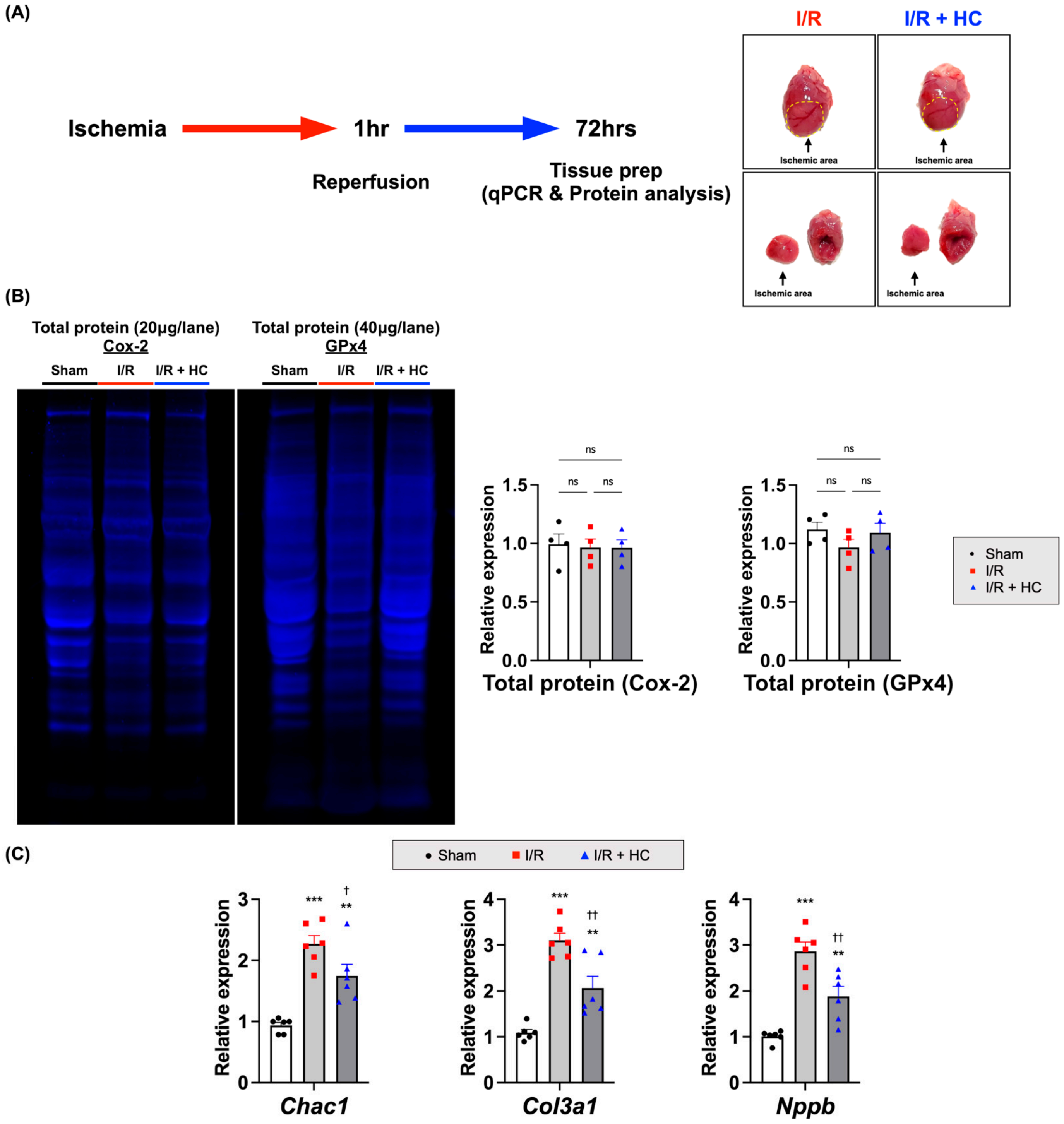

Figure S1. The relative mRNA expression level of ferroptosis related genes after I/R injury. (A) Experimental process of heart tissue sample preparation. All tissue samples obtained from the area at risk (yellow line). (B) The relative images of total protein stain. The total protein was used for normalization of the quantitative analysis. No significant difference among the three groups (n=4) by one-way ANOVA. Error bars show means ± SEM. (C) The relative mRNA expression level of ferroptosis and heart failure related genes using heart tissue harvested 3 days after I/R injury. ** $p < 0.01$; *** $p < 0.001$ versus Sham; † $p < 0.05$; †† $p < 0.01$ versus I/R (n= 6/group) by one-way ANOVA. Error bars show means ± SEM.

Echocardiography (4-weeks)

	Sham (n=5)				I/R (n=5)				I/R + HC (n=6)				Repeated measures Two-way ANOVA P-value
	BASE	1W	2W	4W	BASE	1W	2W	4W	BASE	1W	2W	4W	
LVEF (%)	84.850	87.320	87.320	86.280	61.740	54.430	45.110	39.640	61.591	67.975	71.883	72.233	Sham vs. I/R : <0.001<br>Sham vs. I/R + HC : <0.001<br>I/R vs. I/R + HC : <0.001
LVFS (%)	48.090	51.850	51.820	49.960	29.110	24.590	19.540	16.820	28.716	35.016	36.741	37.100	Sham vs. I/R : <0.001<br>Sham vs. I/R + HC : <0.001<br>I/R vs. I/R + HC : <0.001
SWT (mm)	1.890	1.856	1.856	1.948	1.350	1.178	0.924	0.784	1.323	1.373	1.421	1.358	Sham vs. I/R : <0.001<br>Sham vs. I/R + HC : <0.001<br>I/R vs. I/R + HC : <0.001
LVIDd (mm)	6.609	6.339	6.339	6.572	6.020	7.336	7.986	8.370	5.966	6.346	6.610	7.025	Sham vs. I/R : 0.01<br>Sham vs. I/R + HC : >0.99<br>I/R vs. I/R + HC : 0.01
LVIDs (mm)	3.244	2.922	3.030	3.355	4.272	5.544	6.438	6.974	4.256	4.145	4.201	4.463	Sham vs. I/R : <0.001<br>Sham vs. I/R + HC : 0.005<br>I/R vs. I/R + HC : <0.001

Figure S2. Echocardiographic parameters. Repeated measures two-way ANOVA was used for comparison of heart function within groups (sham - n=5, I/R - n=5, I/R+HC - n=6).

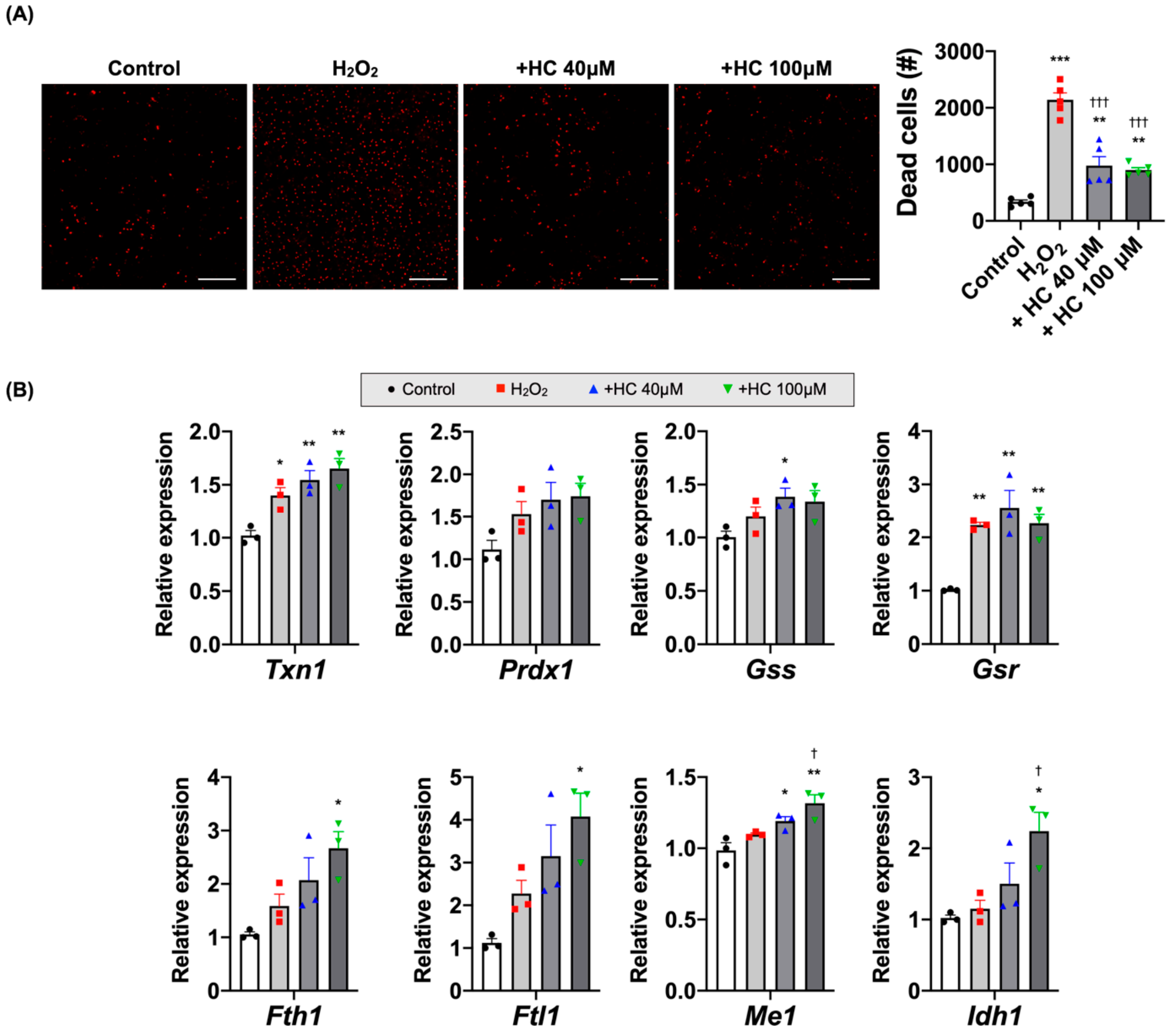

Figure S3. NRCMs treated with H_2O_2, associated with figure 4. (A) The representative images of Live/Dead analysis treated with H_2O_2 and quantitative analysis of dead cell number (red dot). ** $p < 0.01$; *** $p < 0.001$ versus Control; ††† $p < 0.001$ versus H_2O_2 (n= 5/group) by one-way ANOVA. Error bars show means ± SEM. Scale bar = 200 μm. (B) The relative mRNA expression level of antioxidant-related genes in NRCMs treated with H_2O_2. * $p < 0.05$; ** $p < 0.01$; versus Control; † $p < 0.05$; versus H_2O_2 (n= 3/group) by one-way ANOVA. Error bars show means ± SEM.

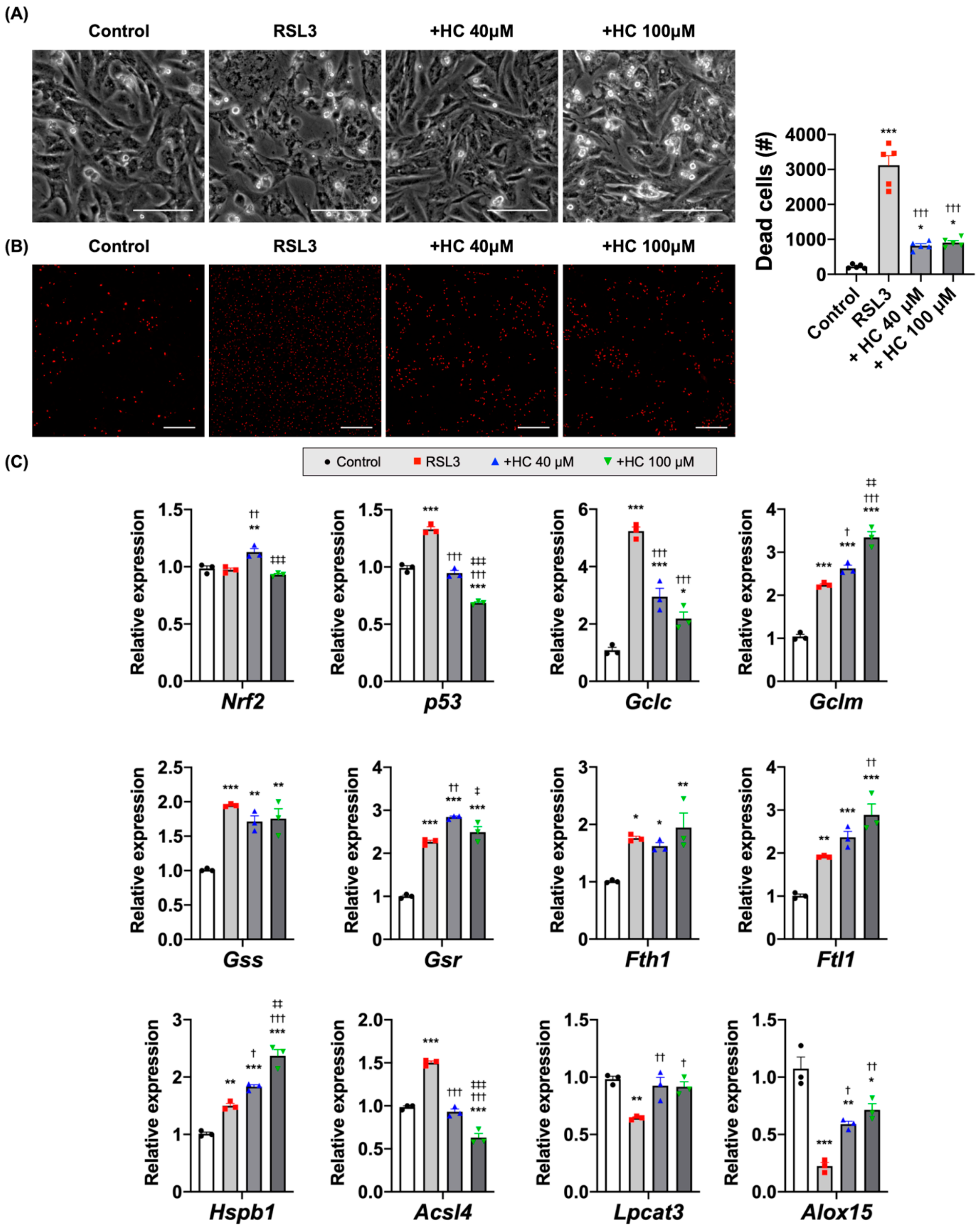

Figure S4. NRCMs treated with RSL3, associated with figure 5. (A) Optical microscope images of RSL3 induced ferroptotic cell death. Scale bar = 100 μm. (B) The representative images of Live/Dead analysis treated with RSL3 and quantitative analysis of dead cell number (red dot). * $p < 0.05$; *** $p < 0.001$ versus Control; ††† $p < 0.001$ versus RSL3 (n= 5/group) by one-way ANOVA. Error bars show means ± SEM. Scale bar = 200 μm. (C) The relative mRNA expression level of ferroptosis-related genes in NRCMs treated with RSL3. * $p < 0.05$; ** $p < 0.01$; *** $p < 0.001$; versus Control; † $p < 0.05$; †† $p < 0.01$; ††† $p < 0.001$; versus RSL3; ‡‡ $p < 0.01$; ‡‡‡ $p < 0.001$ versus +HC 40 μM (n= 3/group) by one-way ANOVA. Error bars show means ± SEM.

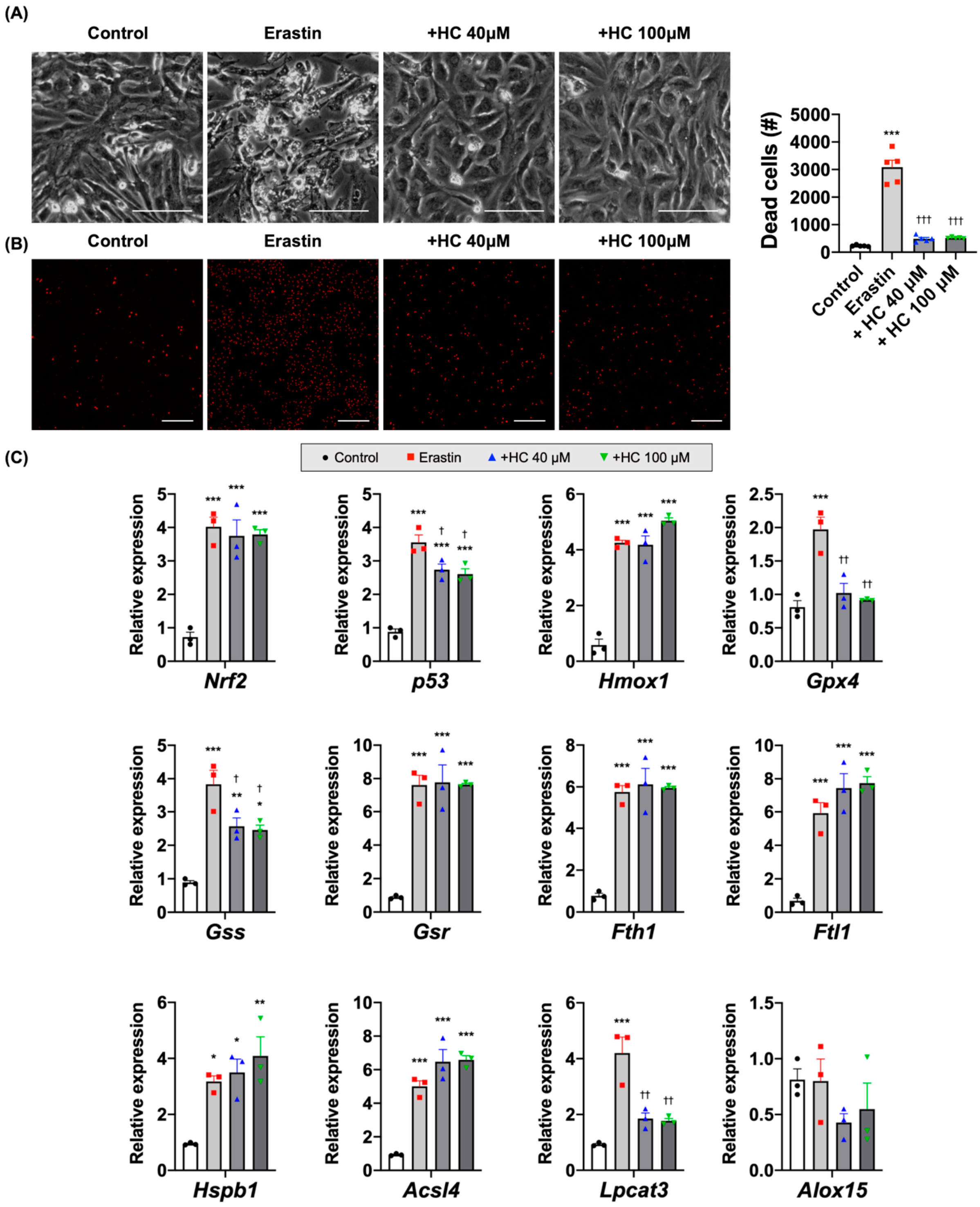

Figure S5. NRCMs treated with erastin, associated with figure 6. (A) Optical microscope images of erastin induced ferroptotic cell death. Scale bar = 100 μm. (B) The representative images of Live/Dead analysis treated with erastin and quantitative analysis of dead cell number (red dot). * $p < 0.05$; *** $p < 0.001$ versus Control; ††† $p < 0.001$ versus Erastin (n= 5/group) by one-way ANOVA. Error bars show means ± SEM. Scale bar = 200 μm. (C) The relative mRNA expression level of ferroptosis-related genes in NRCMs treated with erastin. * $p < 0.05$; ** $p < 0.01$; *** $p < 0.001$; versus Control; † $p < 0.05$; †† $p < 0.01$; versus Erastin (n= 3/group) by one-way ANOVA. Error bars show means ± SEM.

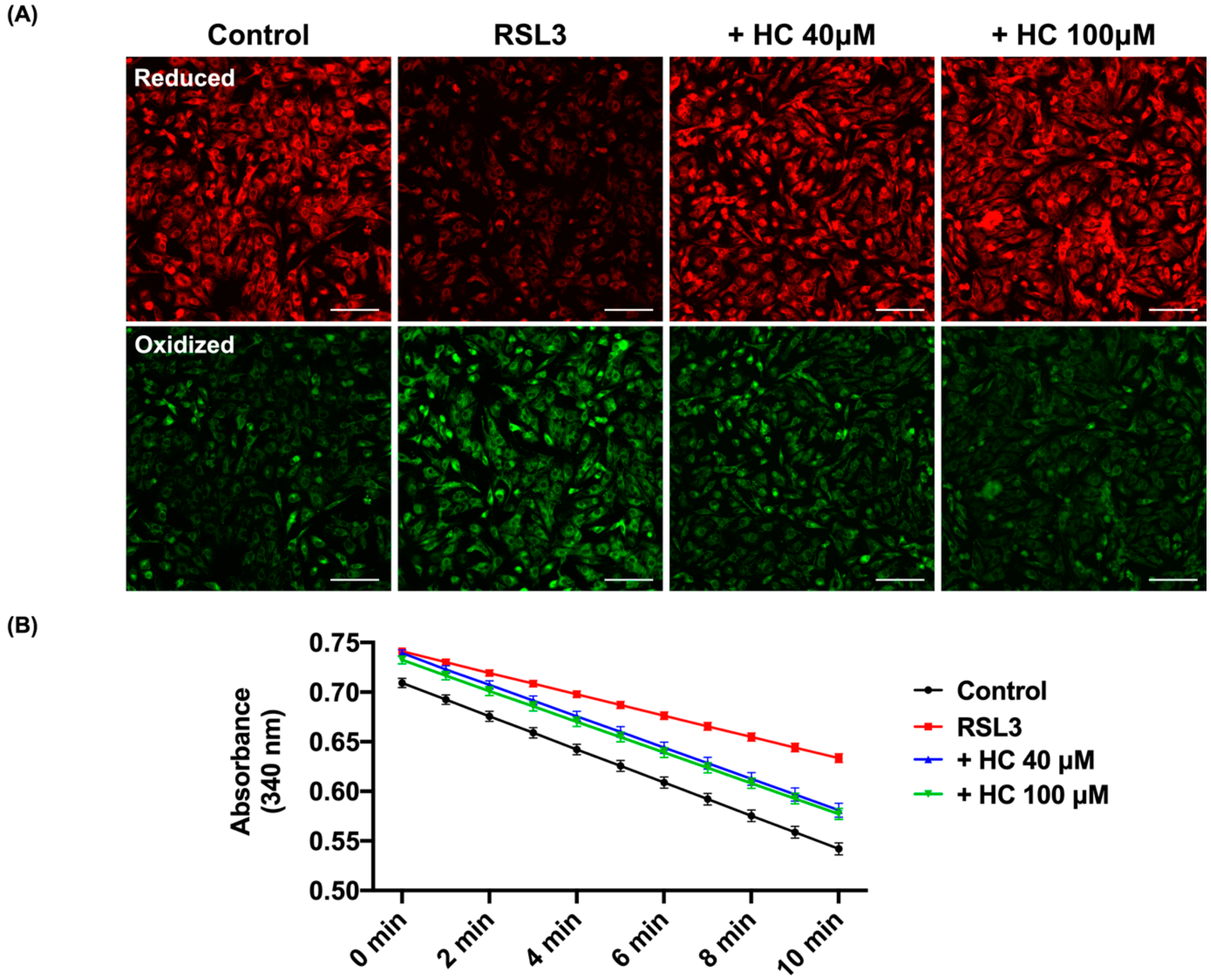

Figure S6. Measurment of lipid peroxidation and GPx activity. (A) The fluorescence of BODIPY 581/581 C11 was measured by the acquisition of green (oxidized) and red (reduced) signals, providing a ratiometric indication of lipid peroxidation. (B) For measurement of GPx activity, the absorbance was read at a wavelength of 340 nm for 10 min at 1 min intervals.

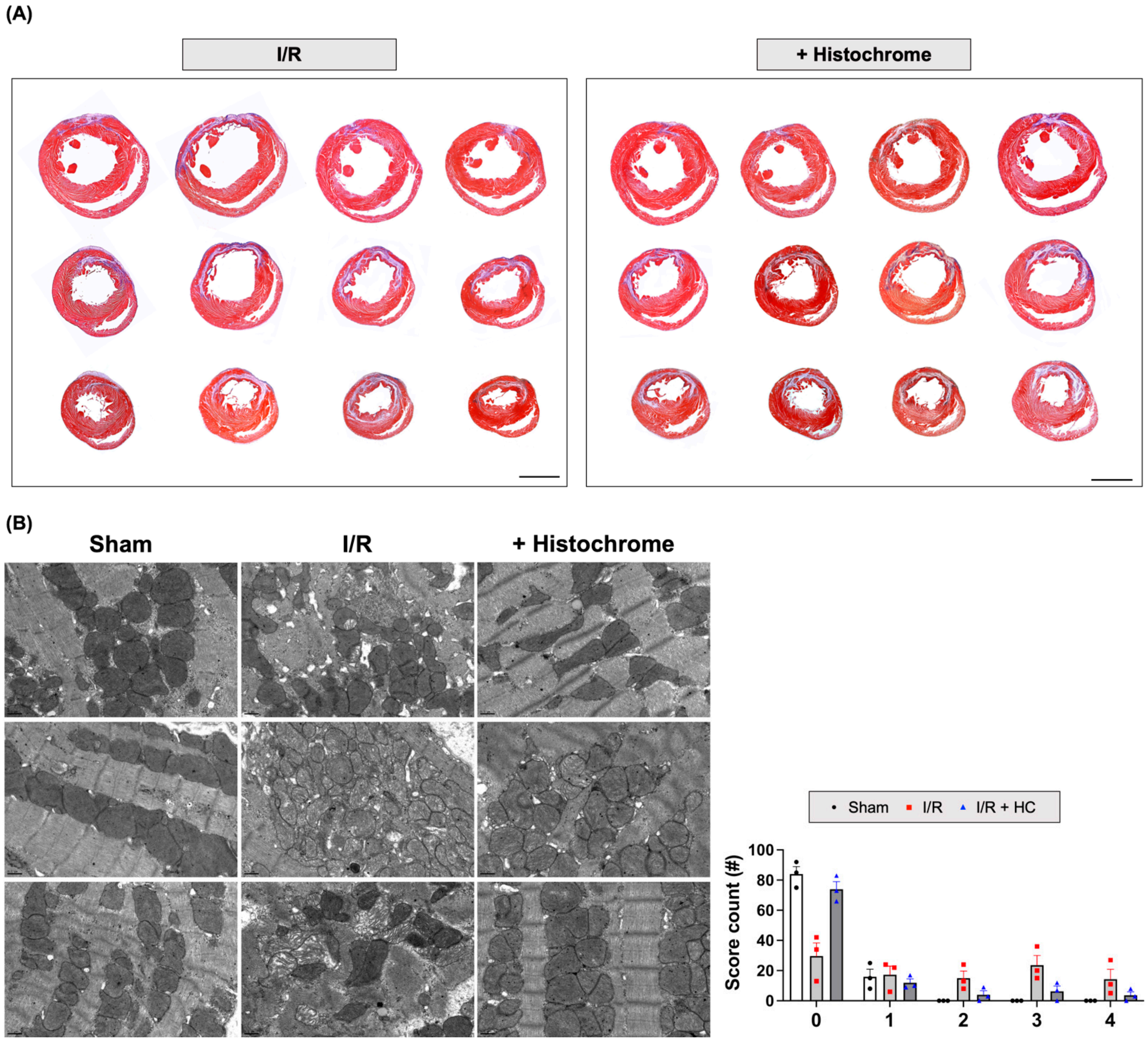

Figure S7. The representative images of Masson's trichrome (MT) staining and evaluation of mitochondrial damage. (A) Representative images of MT stained infarct heart 4 weeks after I/R injury and treated with HC injection. Scale bar = 4000 µm.

(B) The representative transmission electron microscopic (TEM) images of each group. Scale bar = 0.5 µm. Stages of mitochondrial reaction following I/R injury. Score 0 - normal structure with preserved mitochondrial granule, Score 1 - normal structure but granules absent, Score 2 - mitochondrial swelling, Score 3 - disruption of mitochondrial crests, Score 4 - disruption of the crests and loss of integrity of the mitochondrial membrane.

Table S1. Primers used for real-time PCR analysis.

Gene	Primer sequence (5′→ 3′)
Actb **(NM_031144.3)**	
Actb-F	5′-TGT GTG GAT TGG TGG CTC TAT-3′
Actb-R	5′- CAT CGT ACT CCT GCT TGC TGA TC-3′
Hprt **(NM_012583.2)**	
Hprt-F	5′-GCC TAA AAG ACA GCG GCA AG-3′
Hprt-R	5′-GGC CAC AGG ACT AGA ACG TC-3′
Nrf2 **(NM_031789.2)**	
Nrf2-F	5′-GCT ATT TTC CAT TCC CGA GTT AC-3′
Nrf2-R	5′-ATT GCT GTC CAT CTC TGT CAG-3′
Txn1 **(NM_053800.3)**	
Txn1-F	5′-AAG CTG ATC GAG AGC AAG GA-3′
Txn1-R	5′-TTT TGC AAG GTC CAC ACC AC-3′
Txnrd1 **(NM_031614.3)**	
Txnrd1-F	5′-CGG GAG AAG AAG GTT GTC TAT G-3′
Txnrd1-R	5′-GAA CCG CTC TGC TGA GTA AA-3′
Prdx1 **(NM_057114.2)**	
Prdx1-F	5′-CAA AGC CAC GGC TGT TAT GC-3′
Prdx1-R	5′-AGC AAT GAT CTC CGT GGG AC-3′
Slc7a11 **(NM_001107673.3)**	
Slc7a11-F	5′-GGC AAG AAC TCT ACC GTT GGG-3′
Slc7a11-R	5′-GCG TCA TAA ATG TTA GCC CCT G-3′
Aifm2 **(XM_032888506.1)**	
Aifm2-F	5′-GAAAACCATGCGGCAATCTCC-3′
Aifm2-R	5′-CTCCACCAGAGGTTTCTTTCCA-3′
Gclc **(NM_012815.2)**	
Gclc-F	5′- TGA TTG AAG GGA CAC CTG GC-3′
Gclc-R	5′-CGG CGT TTC CTC ATG TTG TC-3′
Gclm **(NM_017305.2)**	
Gclm-F	5′-TCA AGC TCA CAA CTC AGG GG-3′
Gclm-R	5′-CAA ACC ACC ACA TTC ACG CC-3′
Gss **(NM_012962.1)**	
Gss-F	5′-GAA CAA GCA TGT GGG GCA TC-3′
Gss-R	5′-CAC ACA GGG TAG GGG TTG TC-3′
Gsr **(NM_053906.2)**	
Gsr-F	5′-GCC TTC ACC CCG ATG TAT CA-3′
Gsr-R	5′-ATC TCA TCG CAG CCA ATC CC-3′
Gpx4 **(NM_017165.4)**	
Gpx4-F	5′-CGT CTG AGC CGC TTA TTG AAG-3′
Gpx4-R	5′-CAC AGC GCC AAT CAT CGC-3′

***Hmox1* (NM_012580.2)**	
Hmox1-F	5′-GAT GGC CTC CTT GTA CCA TAT C-3′
Hmox1-R	5′-AGC TCC TCA GGG AAG TAG AG-3′
***Fth1* (NM_012848.2)**	
Fth1-F	5′-TGA GCC CTT TGC AAC TTC GT-3′
Fth1-R	5′-CCG AGT CCT GGT GGT AGT TC-3′
***Ftl1* (NM_022500.5)**	
Ftl1-F	5′-TCA CTC TGA AGC ACG ACT AGG-3′
Ftl1-R	5′-CAT TCG GGT GGA GGT GCT GA-3′
***Nqo1* (NM_017000.3)**	
Nqo1-F	5′-ATT GTA TTG GCC CAC GCA GA-3′
Nqo1-R	5′-GAT TCG ACC ACC TCC CAT CC-3′
***Me1* (NM_012600.3)**	
Me1-F	5′-GGA CCC GCA TCT CAA CAA G-3′
Me1-R	5′-CGA AGT CAG AGT TCA GAC GCT-3′
***Idh1* (NM_031510.1)**	
Idh1-F	5′-GGG TGT GAG CGG GGT TAT TG-3′
Idh1-R	5′-TCC TTG CAT TTC CAC CAC AGA-3′
***Ptgs2* (NM_017232.3)**	
Ptgs2-F	5′-ATG CTA CCA TCT GGC TTC GG-3′
Ptgs2-R	5′-TGG AAC AGT CGC TCG TCA TC-3′
***Chac1* (NM_001173437.1)**	
Chac1-F	5′-TGT GGC ATA CCA GGT TCG AG-3′
Chac1-R	5′-TAG TGT CGT AGC CAC CAA GC-3′
***Tp53* (NM_030989.3)**	
Tp53-F	5′-TCC CCT GAA GAC TGG ATA ACT G-3′
Tp53-R	5′-GAC TCA GAG GGA GCT CGA TG-3′
***Acsl4* (NM_053623.1)**	
Acsl4-F	5′-CAC CTT CGA TCC CAG GAG ATT-3′
Acsl4-R	5′-GCG TGA CAG AGC GAT ATG GA-3′
***Lpcat* (NM_001012189.1)**	
Lpcat-F	5′-GGA ATA ATC TGT TTC CCT GGT GGC-3′
Lpcat-R	5′-GGG TGA GGA GTG CTG TGA GAA G-3′
***Alox15* (NM_031010.2)**	
Alox15-F	5′-TCT ACC TGT GGT TGG TTG GAC-3′
Alox15-R	5′-AAT TCT GCT TCC GAG TCC CG-3′
***Hspb1* (NM_031970.4)**	
Hspb1-F	5′-ATC ACT GGC AAG CAC GAA GA-3′
Hspb1-R	5′-GAG CGT GTA TTT CCG GGT GA-3′
***Nppa* (NM_012612.2)**	
Nppa-F	5′-CCT GGA CTG GGG AAG TCA AC-3′
Nppa-R	5′-ATC TAT CGG AGG GGT CCC AG-3′

Nppb **(NM_031545.1)**	
Nppb-F	5′-TTA GGT CTC AAG ACA GCG CC-3′
Nppb-R	5′-CGC CGA TCC GGT CTA TCT TC-3′
Col1a1 **(NM_053304.1)**	
Col1a1-F	5′-GTA CAT CAG CCC AAA CCC CA -3′
Col1a1-R	5′-TCG CTT CCA TAC TCG AAC TGG-3′
Col3a1 **(NM_032085.1)**	
Col3a1-F	5′- AGT GGC CAT AAT GGG GAA CG-3′
Col3a1-R	5′- CAG GGT TTC CAT CCC TTC CG-3′
